# Managing Growth and Dimensionality of Quasi 2D Perovskite Single‐Crystalline Flakes for Tunable Excitons Orientation

**DOI:** 10.1002/adma.202102326

**Published:** 2021-10-08

**Authors:** Marco Cinquino, Antonio Fieramosca, Rosanna Mastria, Laura Polimeno, Anna Moliterni, Vincent Olieric, Naohiro Matsugaki, Riccardo Panico, Milena De Giorgi, Giuseppe Gigli, Cinzia Giannini, Aurora Rizzo, Daniele Sanvitto, Luisa De Marco

**Affiliations:** ^1^ CNR NANOTEC – Institute of Nanotechnology c/o Campus Ecotekne University of Salento Via Monteroni Lecce 73100 Italy; ^2^ Dipartimento di Matematica e Fisica E. De Giorgi Università Del Salento Campus Ecotekne, via Monteroni Lecce 73100 Italy; ^3^ Institute of Crystallography CNR‐IC Via Amendola 122/O Bari 70126 Italy; ^4^ Structural Biology Research Center Photon Factory Institute of Materials Structure Science High Energy Accelerator Research Organization Tsukuba 305‐0801 Japan; ^5^ Swiss Light Source Paul Scherrer Institut Villigen PSI 5232 Switzerland

**Keywords:** crystals synthesis, low‐dimensional perovskites, multiple quantum well, out‐of‐plane excitons, photoluminescence

## Abstract

Hybrid perovskites are among the most promising materials for optoelectronic applications. Their 2D crystalline form is even more interesting since the alternating inorganic and organic layers naturally forge a multiple quantum‐well structure, leading to the formation of stable excitonic resonances. Nevertheless, a controlled modulation of the quantum well width, which is defined by the number of inorganic layers (*n*) between two organic ones, is not trivial and represents the main synthetic challenge in the field. Here, a conceptually innovative approach to easily tune *n* in lead iodide perovskite single‐crystalline flakes is presented. The judicious use of potassium iodide is found to modulate the supersaturation levels of the precursors solution without being part of the final products. This allows to obtain a fine tuning of the *n* value. The excellent optical quality of the as synthesized flakes guarantees an in‐depth analysis by Fourier‐space microscopy, revealing that the excitons orientation can be manipulated by modifying the number of inorganic layers. Excitonic out‐of‐plane component, indeed, is enhanced when “*n*” is increased. The combined advances in the synthesis and optical characterization fill in the picture of the exciton behavior in low‐dimensional perovskite, paving the way to the design of materials with improved optoelectronic characteristics.

## Graphical abstract

Centimeter‐sized quasi 2D‐perovskites with tunable number of inorganic layers are synthesized by an innovative synthetic approach based on the use of a reactant, potassium iodide, that can modulate the supersaturation levels of the perovskite precursors solution without being part of the final products. Exciton orientation is changed from “in‐plane” to “out‐of‐plane” by increasing the “*n*” value.

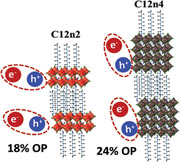

## 1 Introduction

Hybrid organic–inorganic perovskites (PVKs) are in the research spotlight thanks to their outstanding photophysical properties combined with mild synthetic condition, straightforward processability, and tunable optical and electrical properties in function of their structure.^[^ ^1^, ^2^ ^]^ Such unique combination of factors is underpinning their diffusion as key active materials in solar cells,^[^ ^3^ ^]^ light‐emitting diodes,^[^ ^4^ ^]^ photodetectors,^[^ ^5^, ^6^ ^]^ and photonic devices,^[^ ^7^, ^8^, ^9^, ^10^, ^11^, ^12^ ^]^ enabling significant breakthrough that are hard‐to‐reach with other classes of materials.

**Figure 1:**
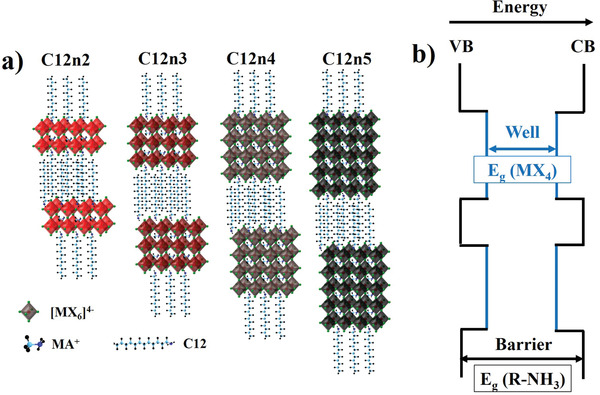
a) Schematic illustration of (C12)_2_(MA)*_n_*_−1_Pb*_n_* I_3_ *_n_*_+1_ (with *n* = 2, 3, 4, and 5) and b) multiple quantum well (MQW) structure.

2D perovskites (2D‐PVKs) represent a promising alternative to the 3D counterparts due to their enhanced environmental stability^[^ ^13^, ^14^, ^15^ ^]^ and larger degrees of structural freedom.^[^ ^2^ ^]^ Ruddlesden–Popper halide perovskites (RPPs) are 2D‐PVKs having the general formula (R‐NH_3_)_2_(A)*_n_*_−1_M*_n_* X_3_ *_n_*_+1_, where R is an alkyl or aromatic moiety, A is a small cation (e.g., methylammonium, MA^+^), M is a divalent metallic cation (such as Pb^2+^) and X is a halide (such as Cl^−^, Br^−^, or I^−^). Their structure consists of inorganic layers of corner‐sharing MX_6_^4−^ octahedra sandwiched between long‐chain organic cations which are linked to MX_6_^4−^ by ionic bond between the ammonium groups and the halide anions. The variable “*n*” indicates the number of MX_6_^4−^ layers stacked between two R‐NH_3_^+^ (**Figure** **1**a).

Since inorganic layers are separated by electronically insulating organic spacers, a multiple quantum well (MQW) structure is naturally formed where inorganic and organic layers act as energetic “well” and “barrier”, respectively (Figure 1b). Spatial (quantum) and dielectric confinement provided by the MQW structure leads to the formation of stable excitons with large binding energies at room temperature (hundreds of meV)^[^ ^2^, ^16^, ^17^ ^]^ and nonlinear optical properties^[^ ^18^ ^]^ appealing for different optoelectronics devices.

The MQW structure can be tailored by tuning the synthetic process, i.e., playing with the nature and amounts of precursors. In particular, the quantum well thickness in RPPs can be adjusted by varying the inorganic layers number “*n*” (i.e., *n *= 1,2,3, etc.) resulting in changes of the bandgap energies, electronic confinement, exciton binding energy,^[^ ^18^ ^]^ and providing an unprecedented opportunity to fully determine the magnitude of in‐plane (IP) and out‐of‐plane (OP) excitonic components and their modulation moving from 2D to 3D systems.

The customization of these systems has interesting implications for the functionality of various optoelectronic devices. For example, it has been observed that changing the number of *n* in (BA)_2_(MA)*_n_*_−1_Pb*_n_* I_3_ *_n_*_+1_ gives multicolor lasing at different wavelengths for *n* > 3 while stimulated emission is inhibited in *n* ≤ 2 members due to enhanced Auger recombination and exciton–phonon interaction (related to the strong quantum confinement).^[^ ^10^ ^]^ These results demonstrate that RPPs are attractive materials for the realization of low‐consumption and low‐cost lasers.

However, although many methods exist for the wet‐chemical synthesis of RPPs having a single inorganic layer (*n* = 1), the preparation of *n* > 1 RPPs homologous is still a big challenge due to the complex equilibrium of ionic species in the precursor's solution. Indeed, the difference in solubility between the alkyl or aromatic organic spacer and methylammonium cation makes difficult the controlled growth of pure phase crystals.^[^ ^19^ ^]^ This is exacerbated in case of larger spacers^[^ ^12^, ^20^, ^21^ ^]^ that more likely are prone to precipitate and to induce the formation of RPPs with *n* = 1 and/or mixed phases.

The few examples of synthetic protocols for *n* > 1 single crystals RPPs, usually based on small organic ligands (such as butylammonium or phenethylammonium, PEA),^[^ ^5^, ^19^, ^22^, ^23^ ^]^ require hot hydroiodic acid to dissolve the precursors and subsequent oversaturation to allow crystal growth. In these approaches, the precursors molar ratio plays a key role in determining the width “*n*” of the inorganic layer: the higher the molar ratio MA^+^/RNH_3_^+^, the thicker the inorganic layer. Unfortunately, the reported protocols are technically challenging since the optimized parameters (i.e., concentration, molar ratio and temperature) significantly change by varying the precursors and are empirically determined from time to time. Moreover, even a small deviation from the optimal concentrations results in the growth of crystals containing impurities consisting of thicker (e.g., *n* + 1) and/or thinner (e.g., *n* − 1) RPPs.^[^ ^23^, ^24^ ^]^ Indeed, the complete understanding of the processes that drive the crystals growth is still missing, consequently it is no possible to predict the optimal and reproducible synthetic conditions.

Here, we propose an innovative synthetic concept for RPPs that allows to tune the quantum well width “*n*” by modulating the crystallization rate through the control of supersaturation level of the precursors solution. This approach exploits an iodide salt as additive that, acting as an additional source of I^−^, induces the generation of iodoplumbate species and results into an increase of the monomer density. Differently from other methods, our protocol allows to fix the molar ratio of PbI_2_, MA^+^, and RNH_3_^+^ and to control the “*n*” value by simply modulating the amount of potassium iodide (KI) added in the precursors solution.

The effectiveness of this method is demonstrated by the production of centimeter‐sized, dodecylammonium lead halide RPPs having the formula (C12)_2_(MA)*_n_*_−1_Pb*_n_* I_3_ *_n_*_+1_ (C12 = dodecylammonium, C_12_H_25_NH_3_^+^) with *n* = 2, 3, 4, and 5 (Figure 1a) some of them presented here for the first time. It is worth to underline that the as developed synthetic procedure overcomes organic precursor solubility issues and thus allows the introduction of a very long alkyl chain organic spacer, such as dodecylammonium, which is sought to enhance stability under environmental conditions (air, humidity).^[^ ^15^, ^25^ ^]^ In addition, by increasing the organic carbon chain length, the softness of RPPs can be tuned and the structural flexibility could be enhanced,^[^ ^25^ ^]^ enabling the use of these hybrid semiconductors in flexible, wearable electronic devices, an unexplored field for perovskite materials. Moreover, the energy and confinement of excitons can be tailored by introducing unconventional organic spacers, and the synthesis of high‐n RPPs based on them represents a unique opportunity to unlock new functionalities of perovskites and to explore the new related physics.^[^ ^26^ ^]^

We exploit as‐prepared RPPs as platform to investigate by Fourier‐resolved polarized photoluminescence (PL) the dipole orientation of the excitons in low dimensional systems, finding that the out‐of‐plane excitonic component is increased with the thickness of the quantum well.

These results expand the knowledge on the chemistry involved in the complex synthesis of these hybrid semiconductors and on the fundamental physical properties of RPPs, allowing to correlate their structure with their variable exciton anisotropy that could be widely exploited in polarization‐resolved optical devices.

## 2 Results and Discussion

### 2.1 Synthesis of 2D‐PVKs Single‐Crystalline Flakes

**Figure 2:**
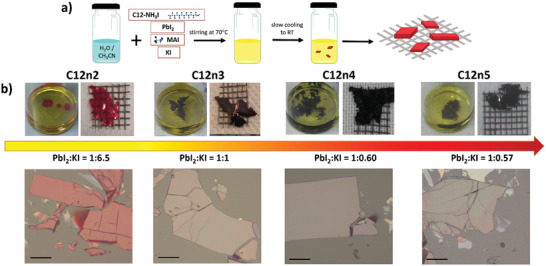
a) Schematic illustration of new potassium iodide controlled synthesis. b) Photographs of 2D‐PVKs single‐crystalline flakes (scale bar: 100 µm).

(C12)_2_(MA)Pb_2_I_7_, (C12)_2_(MA)_2_Pb_3_I_10_, (C12)_2_(MA)_3_Pb_4_I_13_, and (C12)_2_(MA)_4_Pb_5_I_16_ having *n* = 2, 3, 4, and 5 henceforth named C12n2, C12n3, C12n4, and C12n5, respectively, are prepared using a new one‐step synthetic approach in which lead iodide (PbI_2_), *n*‐dodecylammonium iodide (C_12_H_25_NH_3_I), methylammonium iodide (CH_3_NH_3_I or MAI), and KI are dissolved in water/acetonitrile mixture. The resulting bright yellow solution is then left undisturbed in a closed vial in ambient conditions. The slow cooling down to room temperature induces supersaturation, which is driven by the reduced solubility at lower temperature of the precursors (primarily C12), and the subsequent formation of small nuclei at the bottom of the vial (after about 1 h). At the end of the process, cm‐sized platelets are picked up with the help of a net and gently dried with paper towel in order to remove residual reactant solution (see sketch of the process in **Figure** **2**a).

As‐grown crystals show lamellar topography (see Figure 2b, top) arising from the randomly stacked flakes at the centimeter‐scale. The color of the crystals darkens with higher *n*, due to the narrowing of the bandgap, going from bright red (C12n2) to dark red (C12n3) and black (C12n4 and C12n5). Crystals morphology is visible from microscope images (Figure 2b, bottom).

**Figure 3:**
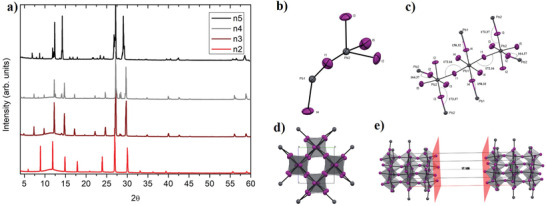
a) X‐ray diffraction patterns of C12n2, C12n3, and C12n4 RPPs. b) A view of the asymmetric unit with the atomic labeling scheme of (C12)_2_(MA)_2_Pb_3_I_10_ (*n* = 3) and a view of the local environment of the asymmetric unit showing the polyhedral coordination of the Pb atoms and the distortion angles (in °) of the inorganic layers (see Table S3, Supporting Information). Ellipsoids are drawn at 50% of probability level. d) A view of (C12)_2_(MA)_2_Pb_3_I_10_ (*n* = 3) along *a* of the crystal packing and e) a view of the crystal packing showing the distance (in Å) between the two nearest slabs of triplets of inorganic layers.

X‐ray diffraction (XRD) patterns of C12n2, C12n3, C12n4, and C12n5 perovskites are shown in **Figure** **3**. All crystals show characteristic stacking peaks of 2D layered materials. In particular, below 14° 2θ angle we observe three, four, five, or six reflections for crystals having *n* = 2, 3, 4, and 5, respectively (Figure 3a). The appearance of additional peaks with the increase of “*n*” indicates the presence of additional inorganic layers along the stacking direction.^[^ ^19^, ^27^, ^28^ ^]^

Synchrotron single‐crystal X‐ray diffraction has been performed on the synthesized materials. Given the peculiarity of the samples consisting of randomly stacked thin flakes and the well‐known difficulty in isolating single crystals of suitable quality and/or phase‐pure high *n*‐member RPPs^[^ ^24^, ^25^, ^29^ ^]^ we refined the crystal structure of *n* = 2 and *n* = 3 RPPs. Crystallographic details on the structure refinement are supplied in Figure 3b–e and Figures S1 and S2 and Tables S1–S3 in the Supporting Information.

In our synthetic approach, differently from all the other reported methods, the PbI_2_:MAI:C12 ratio is kept fixed while the amount of KI in solution is tuned to finely vary the thickness of the inorganic layer (value of “*n*”) accordingly. Namely, the higher the KI concentration, the lower the value of “*n*.” In particular, C12n2, C12n3, and C12n4 are obtained by using precursor solution having Pb:KI ratio of 1:6.5, 1:1, and 1:0.6, respectively.

The precursors PbI_2_, MAI, and C12 are deliberately used in nonstoichiometric molar ratio, 1/4.5/0.025 (see Table S4, Supporting Information, upper section and experimental section for more details) taking into account the low solubility of long alkyl chain cations in polar solvent and their tendency to precipitate faster than MAI, driving the synthesis toward *n* = 1 RPPs, as already reported.^[^ ^3^, ^19^, ^23^ ^]^ Thus, to regulate the precipitation rates, a low amount of C12 and an excess of MAI are used in the starting mixture. With our method it is possible to introduce a dodecyl carbon chain which is twice the longest organic molecule used so far in *n* > 1 RPPs, providing the synthesized perovskites with a more hydrophobic character.

The above reported results indicate that the concentration of KI is the key parameter for the fine modulation of the inorganic layer thickness in RPPs with high phase purity.

To deepen the understanding on the chemistry underlying the growth mechanism, we investigate the solution chemistry and complexation equilibria of all the involved chemical species by performing UV–vis absorption measurements of the precursors solution containing different amount of KI. The spectra (see Figure S3, Supporting Information) show three peaks at 262, 314, and 355 nm attributable to Pb^2+^ ions, PbI_2_, and PbI_3_^−^ iodoplumbate complex respectively.^[^ ^28^ ^]^ By increasing the concentration of KI in solution, the peaks related to PbI_2_, PbI_3_^−^ increase accordingly revealing the increasing concentration of iodoplumbate complexes in solution.

In our approach, KI provides I^−^ ions which promote the dissolution of PbI_2_, poorly soluble in water/acetonitrile mixture, and induce the formation of iodoplumbate complexes^[^ ^30^, ^31^, ^32^ ^]^ allowing to reach supersaturation of the monomers in the precursor solution. Since the level of supersaturation strongly influence the nucleation rate, by modulating the amount of KI added to the precursor solution, it is possible to induce a fast or slow nucleation of low‐*n* or high‐*n* RPPs crystals, respectively. The higher the KI concentration, the faster will be the formation of low‐*n* crystals; we observe indeed that precipitation of *n* = 2 large crystals occurs in only 3 h. On the other hand, solutions containing lower amount of KI are characterized by a lower supersaturation degree which results in a strong slowdown of the nucleation and growth (8 h for *n* = 3 and 16 h for *n* = 4 and *n* = 5) promoting the evolution of high *n* RPP crystals.

To further investigate the role of I^−^ to control nucleation and growth of RPPs crystals, we modulate the concentration of MAI maintaining fixed the concentration of PbI_2_, C12, and KI (see Table S4, Supporting Information, lower section “Modulation of PbI_2_/MAI ratio”). We found that MAI, providing additional I^−^ to the solution in a similar way to KI, can affect the growth as well. However, although we are able to synthesize *n* = 2 and *n* = 3 crystals we cannot obtain pure perovskite phase when *n* is >3 but only *n* = 4 and *n* = 5 mixed perovskite phases.

This can be ascribed to the different role that KI and MAI have in the growth of the crystal: while KI only influence the concentration of iodoplumbate complexes, regulating supersaturation and hence the growth of perovskite crystals, MAI both induce the formation of iodoplumbate and takes part to the perovskite structure, indeed, the variation of the levels of MAI in solution is more critical and even a small deviation greatly affect the stoichiometry of the growing perovskite crystals.

Given the delicate crystallization equilibrium of these hybrid materials, the great novelty of our approach consists in the introduction of a reactant that contributes to modulate the supersaturation levels without being part of the final products: in this way the variation of the KI amounts does not alter the structure of the desired perovskite. Moreover, as additional advantage, our synthetic procedure requires less steps and milder conditions with respect to other methods.^[^ ^19^, ^23^, ^25^ ^]^

To demonstrate the effectiveness of this approach irrespective to the chemical nature of the organic spacer, we use PEA as organic layer instead of C12. Despite the completely different chemical structure of PEA we observe the same trend: with high concentration of KI in the solution we obtain *n* = 2 crystals, whereas by lowering the concentration of KI we are able to synthetize *n* = 4 RPPs (see Table S5 and Figure S4, Supporting Information).

### 2.2 Optical Characterization of 2D‐PVKs Single‐Crystalline Flakes

**Figure 4:**
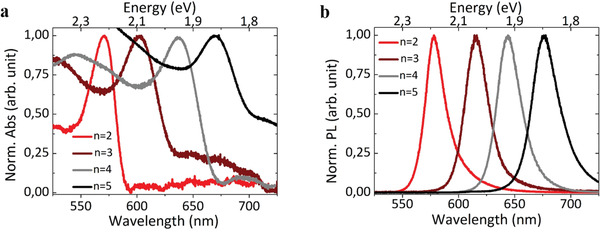
a) Abs and b) PL spectra of C12n2, C12n3, C12n4, and C12n5 single‐crystalline flakes at room temperature.

Room temperature absorption (Abs) and PL spectra acquired on exfoliated C12n2, C12n3, C12n4, and C12n5 are shown in **Figure** **4**a,b, respectively.

As the thickness of the inorganic layer *n* increases from 2 to 5, a redshift in both Abs and PL excitonic resonance is observed: in particular, PL spectra have peaks centered at 582 nm for *n* = 2, 618 nm for *n* = 3, 646 nm for *n* = 4, and 678 nm for *n* = 5. This is because as the width of the quantum well increases, the electronic bandgap decreases due to the reduced quantum confinement, as already reported for these materials.^[^ ^5^, ^19^, ^23^, ^25^ ^]^ PL spectra show narrow FWHM (full width at half maximum), consistent with the crystalline nature and Stokes shifts of about 10 nm (see **Table** **1**). Abs and PL measurements are performed on different regions of the same flake and on different crystals, revealing the presence of single peaks only for *n* = 2, *n* = 3, and *n* = 4 RPPs, highlighting the good quality of these materials. C12n5 consists of a dominant phase which has a single PL peak at 678 nm, although, by scanning throughout the crystals we detected the presence of small and rare *n* > 5 domains (see Figure S5, Supporting Information), which is quite common for high‐*n* members of RPPs where different phases easily cocrystallize.^[^ ^24^, ^25^ ^]^

**Table 1:** Summary of absorption (Abs) peak wavelengths, photoluminescence (PL) peak wavelengths, PL full width at half maxima (FWHM), and Stokes shifts of (C12)_2_(MA)*_n_*_−1_Pb*_n_* I_3_ *_n_*_+1_ (*n* = 2, 3, 4, and 5) single‐crystalline flakes at room temperature

Sample	Abs peak wavelength [nm]	PL peak wavelength [nm]	PL FWHM [nm]	Stokes shift [nm]
*n* = 2	571 ± 1	582 ± 1	26 ± 1	11 ± 2
*n* = 3	603 ± 1	618 ± 1	27 ± 1	15 ± 2
*n* = 4	636 ± 1	646 ± 2	28 ± 1	10 ± 3
*n* = 5	670 ± 1	678 ± 2	31 ± 1	8 ± 3

Abs and PL spectra taken on phenethylammonium iodide (PEAI) *n* = 2, *n* = 3, and *n* = 4 homologous (see Figures S6 and S7, Supporting Information) show the same behavior, demonstrating that the organic layer can induce a slight shift on the excitons energy in RPPs.

The layered crystal structure of 2D perovskite results in an intrinsic anisotropy, which reflects in anisotropic optical properties, such as refractive index and dipole orientation.

In general, going from *n* = 1 toward a bulk material (*n* = ∞), the dipole orientation should change from an almost purely IP to an isotropic orientation. A gradual increase of the OP component by increasing *n* is expected to be clearly visible in crystals which are not affected by disorder, grain‐to‐grain heterogeneity or tilted domains that could randomize the orientations, as typically observed in spin coated RPP thin films.^[^ ^33^ ^]^

Here, we take advantage of the good optical quality of RPP flakes to demonstrate the dipole orientation by using Fourier‐resolved polarized photoluminescence, which relies on the study of the PL intensity pattern as a function of the in‐plane momentum *k*, for s and p polarizations. In particular, IP oriented dipoles contribute to the local density of optical states (LDOS) of both polarizations, while OP oriented dipoles contribute only to the LDOS of p polarization. In order to completely distinguish between the IP and OP contributions in p polarization, we have carried out the experiments at a specific angle, the total internal reflection angle (TIR), at which the IP contribution is totally suppressed, allowing to capture only the OP component.

The Fourier‐resolved polarized photoluminescence technique requires ultrathin crystals. For this reason, we mechanically exfoliate the synthesized crystals to obtain flakes having a thickness <50 nm. We collect the PL signal lying beyond the TIR angle employing an oil immersion microscope objective to extract the confined signal (see the Experimental Section). In order to fit the experimental data, we used the analytical model reported by Schuller et al.^[^ ^34^ ^]^

**Figure 5:**
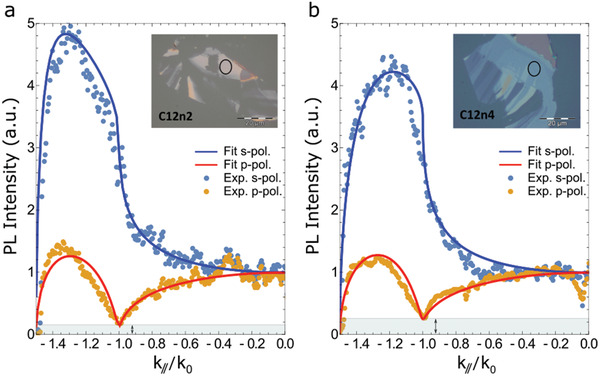
Fourier‐resolved photoluminescence measurements. a) PL intensity as a function of the normalized in‐plane wavevector (*k*_//_/*k*_0_) for C12n2 crystal. The light blue and orange dots represent the experimental data for s and p polarization, respectively. Blue and red solid lines indicate the fit of the experimental data for s and p polarization, respectively. b) PL intensity as a function of the normalized in‐plane wavevector (*k*_//_/*k*_0_) for C12n4 crystals. The gray box and black arrow, visible in the bottom part of the figures, underline the different amplitude of the dip visible at the TIR angle for p polarization for the two cases. The inset shows the real space of the measured crystals, where the black circle indicates the position of the excitation spot.

The experimental (dots) and analytical (solid lines) PL intensity profiles as a function of the normalized in‐plane wavevector (*k*_//_/*k*_0_) for both s (light blue dots and blue solid line) and p (orange dots and red solid line) polarizations are shown in **Figure** **5**a,b for C12n2 and C12n4 crystals, respectively. Here we show the PL profiles for the negative portion of the normalized in‐plane wavevector, but the profiles are symmetric in *k*. The selected crystals are shown in the insets of the two figures where the black circle indicates the position of the excitation laser.

At the TIR angle, the p polarization PL profile presents a sharp modulation, whose depth is related to the OP component: higher is the modulation depth, lower is the OP component. The Fourier‐resolved photoluminescence experiments performed on C12n2 and C12n4 crystals (see Figure 5a,b) clearly show that the OP contribution is different in the two cases (as highlighted by the width of the gray regions in Figure 5a,b and the black arrows). In particular, from the fitting of the experimental data we obtain an OP component of 18% ± 3% for *n* = 2, while the contribution increases up to 24% ± 4% for *n* = 4 perovskite.

The limited thickness of the well of C12n2 (1.2 nm thick) confines the majority of the excitonic wavefunction within two lead halide octhaedra layers, but still a small amount of OP oscillating dipoles is visible. This observation is in good agreement with previous experimental evidences,^[^ ^35^, ^36^ ^]^ which have shown that a small fraction of OP dipole is presents even for the *n* = 1 case. When *n* increases to four layers (2.4 nm thick), we observe a significant enhancement of the OP fraction, revealing that the OP excitonic component oscillating in the normal direction with respect to the plane of the perovskite crystal becomes more prominent. To properly frame these values, we have to compare them both with a completely isotropic system (randomly oriented organic molecules) and with a completely 2D system such as MoS_2._ Taking into account that a fully IP excitons system (MoS_2_ monolayer) shows <5% OP contribution while an isotropic emitter has an OP fraction of 33%,^[^ ^35^ ^]^ we can assert that a significant *n*‐dependent variation of the OP contribution is demonstrated in our quasi‐2D systems.

In Figures S8 and S9 in the Supporting Information we show the results obtained for *n* = 2, *n* = 3, *n* = 4, and *n* = 5 single‐crystalline flakes, which show a continuous decrease of the % IP component as *n* increases. Thanks to this technique and to the high optical quality of the crystals, we obtained detailed information on the quantitative excitonic components (in‐plane and out‐of‐plane), so that we can easily identify a clear trend of such contributions in *n* = 2, 3, 4, and 5 perovskites (Figure S9, Supporting Information).

These results show that the IP and OP excitonic components in RPPs could be precisely tuned by engineering the quantum well architecture. This opens new opportunities for the design of optoelectronic devices able to exploit polarizable emission. Indeed, by further engineering the architecture of the crystals, they could sustain the formation of dipolar‐excitons^[^ ^37^, ^38^ ^]^ which possess superior nonlinear optical properties to be used in polaritonic devices.

## 3 Conclusions

In summary, centimeter‐sized 2D‐PVKs single‐crystalline flakes (*n* = 2, 3, 4, and 5) have been successfully synthesized through a conceptually new acid‐free solution growth technique. The keystone of the proposed approach consists in the fine control of crystallization rate through the modulation of the supersaturation level of the precursor solution mediated by the addition of potassium iodide as additive. By exploiting this approach, the thickness of the inorganic layer has been finely tuned and the effectiveness of the methods has been demonstrated for different organic spacers, namely PEA and C12.

Perovskites obtained with this method exhibit good optical properties that allow to reveal different excitonic contributions that changes from “in‐plane” to “out‐of‐plane” by increasing the “*n*” value.

This work provides new insights into the chemistry involved in the complex synthesis of these hybrid semiconductors and offers rational guidelines for the development of a general synthetic strategy for tuning the thickness of inorganic layers, enriching the family of RPPs to be applied in optoelectronic devices.

## 4 Experimental Section

### Chemicals and Reagents

Lead(II) iodide (PbI_2_), ultradry (99.999% metals basis) was purchased from Alfa Aesar. Methylammonium iodide, phenethylammonium iodide, and *n*‐dodecylammonium iodide were acquired from Greatcell Solar, Acetonitrile was purchased from Sigma‐Aldrich. All salts and solvents were used as received without any further purification.

### Synthesis

All syntheses were carried out in air and in a fume hood. 447 mg of lead iodide (PbI_2_), 7.4 mg of *n*‐dodecylammonium iodide (C_12_H_25_NH_3_I), and 693.9 mg of methylammonium iodide (CH_3_NH_3_I) were dissolved at 70 °C in a mix of water/acetonitrile (1.05/1 mL respectively). Different amounts of potassium iodide (1045 mg for *n *= 2, 161 mg for *n *= 3, 99.6 mg for *n *= 4, 93 mg for *n *= 5, respectively) were added to the solution to control the value of *n*. All the precursors were stirred at 70 °C for 1 h producing a bright yellow solution. Perovskite single‐crystalline flakes were then grown by storing the bright solution at room temperature. After 1 h at room temperature, small nuclei appear at different positions at the bottom of the vial. Then they started to grow into large crystals. After some hours (3, 8, 16, and 16 for *n* = 2, *n* = 3, *n* = 4, and *n* = 5, respectively), crystals at the bottom of the vial became thick and cm‐sized platelets.

Lead iodide 447 mg, PEAI (C_8_H_9_NH_3_I) 46 mg, methylammonium iodide 370 mg, and potassium iodide (1400 mg for *n* = 2, 1000 mg for *n* = 3, and 875 mg for *n* = 4) were dissolved at 70 °C in a mix of water/acetonitrile (1.05/0.45 mL, respectively). All the precursors were stirred at 70 °C for 1 h producing a bright yellow solution. Then the temperature was raised to 83 °C. After some minutes the crystallization occurs at the water−air interface followed by a fast lateral growth within the water−air plane.

Crystals were extracted using a net and then gently collected and dried with paper in order to remove the remaining perovskite solution. Finally, crystals were carefully stored in the glovebox.

### X‐Ray Diffraction

Single‐crystal X‐ray diffraction data measurements were carried out at the beamline BL‐5A at the Photon Factory, Japan, using a PILATUS3 6M detector. Data collection was performed at room temperature on a selected crystal mounted on litholoops (Molecular Dimensions). Complete data were obtained in a single 360° ω scan in steps of 0.2° at speeds of 2° s^−1^ using a beam energy of 16.53067 keV, λ = 0.75 Å, focus size 75 × 75 µm^2^.

Diffraction data were analyzed by XDS^[^^39]^ a software consisting of eight subroutines able to carry out the main data reduction steps; the process of scaling and correction for absorption effects of the integrated intensities was performed by the XSCALE subroutine.^[^ ^39^ ^]^ Structure solution was carried out by Direct Methods^[^ ^40^ ^]^ using SIR2019^[^ ^41^ ^]^ due to the large structural disorder, only the heavy atoms were reliably located by SIR2019. The partial structure model was anisotropically refined by SHELXL2014/7.^[^ ^42^ ^]^ The programs used to prepare material for publication were WinGX^[^ ^43^ ^]^ and publCIF^[^ ^44^ ^]^ the software Mercury^[^ ^45^ ^]^ was applied for molecular graphics.

Powder X‐ray diffraction was carried out using an X'Pert PRO MRD diffractometer, with a Cu*K*_α_ radiation; the diffraction patterns were acquired in the interval between 2θ = 3° and 60° with a step size of 0.0098° 2θ and a counting time of 100 s.

### Optical Measurements

PL measurements are performed in reflection configuration. A 405 nm CW (continuous‐wave) laser (Vortran Laser Tech.) is used to nonresonantly excite the material through a 10X objective (Olympus, N.A. = 0.25), the same objective is used to collect the PL signal. The detected signal is focused, by using a 30 cm lens, into a 300 mm spectrometer (Princeton Instruments, Acton Spectra Pro SP‐2300) coupled to a charge‐coupled device (Princeton Instruments, Pixies 400). The spectrometer is equipped with three gratings, 150, 300, and 1200 g mm^−1^, all of them blazed at 500 nm. The 300 g mm^−1^ grating is used for PL measurements. A 450 nm cutoff filter (AH511, Newport) along the detection line cuts the residual excitation laser intensity. Absorption measurements are performed in transmission configuration, using a Xenon light source (Korea Spectral Products‐ASB‐XE‐175). The incident white light arrives on the sample through a 10X objective (Rolyn‐Rou, N.A. = 0.30) and is detected by the same optical path used for the PL measurement. Fourier‐resolved polarized photoluminescence measurements are performed by using a 488 nm CW laser with an excitation spot of 2 µm. An oil immersion microscope objective (Olympus, N.A = 1.49) was used to collect the PL signal located beyond the critical angle. A half‐wave plate (AHWP10M‐600) and a linear polarizer (LPVISSE100) were placed in front of the spectrometer in order to resolve the two s and p polarization components. UV–vis absorption spectra are recorded with a double beam spectrophotometer Varian Cary 300 at each addition of KI employing quartz cuvettes with an optical path of 1 cm.

## Conflict of Interest

The authors declare no conflict of interest.

## Supporting information

Supporting information

## Data Availability

The data that support the findings of this study are available from the corresponding author upon reasonable request.
