# Supplementary material for: Managing Growth and Dimensionality of Quasi 2D Perovskite Single‐Crystalline Flakes for Tunable Excitons Orientation
Source: Adv Mater. 2021 Oct 8;33(48):2102326. doi: 10.1002/adma.202102326 (PMC11469044; doi:10.1002/adma.202102326)
Supplement: Supplementary file 1 — Supporting information [file ADMA-33-2102326-s001.pdf]

ADVANCED MATERIALS

Supporting Information

for *Adv. Mater.,* DOI: 10.1002/adma.202102326

Managing Growth and Dimensionality of Quasi 2D Perovskite Single-Crystalline Flakes for Tunable Excitons Orientation

Marco Cinquino, Antonio Fieramosca, Rosanna Mastria, Laura Polimeno, Anna Moliterni, Vincent Olieric, Naohiro Matsugaki, Riccardo Panico, Milena De Giorgi, Giuseppe Gigli, Cinzia Giannini, Aurora Rizzo, Daniele Sanvitto,* and Luisa De Marco**

Supporting Information

Managing Growth And Dimensionality of Quasi 2D Perovskite Single-Crystalline Flakes For Tunable Excitons Orientation

Marco Cinquino (1,2), Antonio Fieramosca (1), Rosanna Mastria (1), Laura Polimeno (1,2), Anna Moliterni (3), Vincent Olieric (4,5), Naohiro Matsugaki (5), Riccardo Panico (1,2), Milena De Giorgi (1), Giuseppe Gigli (1,2), Cinzia Giannini (3), Aurora Rizzo (1), Daniele Sanvitto (1)*, Luisa De Marco (1)**

M. Cinquino, R. Mastria, A. Fieramosca, L. Polimeno, R. Panico, M. De Giorgi, G. Gigli, A. Rizzo, D. Sanvitto, L. De Marco
CNR NANOTEC – Institute of Nanotechnology, c/o campus Ecotekne, University of Salento, Via Monteroni, 73100 Lecce, Italy

M. Cinquino, L. Polimeno, R. Panico, G. Gigli
Dipartimento di Matematica e Fisica E. De Giorgi, Università Del Salento, Campus Ecotekne, via Monteroni, Lecce, 73100, Italy

A. Moliterni, C. Giannini
Institute of Crystallography, CNR-IC, Via Amendola 122/O, 70126, Bari, Italy

V. Olieric, N. Matsugaki
Structural Biology Research Center, Photon Factory, Institute of Materials Structure Science, High Energy Accelerator Research Organization, Tsukuba, 305-0801, Japan

V. Olieric
Swiss Light Source, Paul Scherrer Institut, Villigen PSI, 5232, Switzerland

E-mail: rosanna.mastria@nanotec.cnr.it, daniele.sanvitto@nanotec.cnr.it, luisa.demarco@nanotec.cnr.it

Synchrotron single-crystal X-ray diffraction

$(DA)_2(MA)Pb_2I_7$ (n = 2) and $(DA)_2(MA)_2Pb_3I_{10}$ (n = 3) crystallized in the space centrosymmetric group *Pnma* and $P2_1/c$, respectively; both space groups were determined by *SIR2019*, by assuming the Laue group compatible with the crystal system to which the compounds belong and calculating, for each possible extinction symbol, a probability value *via* a statistical analysis based on the experimental intensities.

At the end of the structure solution process *SIR2019* provided a partial model consisting of heavy atoms only, because, due to the structural disorder, the light atoms of the organic chains were not located. The heavy atoms were anisotropically refined by using full-matrix least-squares techniques by *SHELXL2014/7*. The knowledge of the heavy atoms positions allowed to have insights into the crystal packing and evaluate the distortion of the inorganic chains.

Main crystallographic data are provided in Table S1; additional tables (*i.e.*, Tables S2 and S3 for $(DA)_2(MA)Pb_2I_7$ (n = 2) and $(DA)_2(MA)_2Pb_3I_{10}$ (n = 3), respectively) supply refined fractional atomic coordinates, displacement parameters, bond distances and angles.

$(DA)_2(MA)Pb_2I_7$ (n = 2) has been recently characterized [1]; the published crystal structure crystallized in the space group $P4_2/n$ with cell parameters $a_T = b_T$ =8.899(3), c_T = 59.570(4) Å. The compound $(DA)_2(MA)Pb_2I_7$ (n = 2) studied in this work was orthorhombic, cell parameters a = 8.853(2), b =59.264(3) and c =8.933(3) Å, with $a \approx c \approx a_T$ and $b \approx c_T$; the cell was pseudo-tetragonal and the space group (*i.e.*, *Pnma*) was different from the space group of the published structure [1], consequently, the $(DA)_2(MA)Pb_2I_7$ (n = 2) compound characterized in the present work is a new polymorph. Figure S1 shows a view of the asymmetric unit (a) and its local environment (b), together with the values of some bond angles listed in Table S2. The bridging in-plane distortion angle Pb–I–Pb angle was 166.33° (see Figure S1 and Table S2), far from 180.0° (*i.e.*, the typical value of undistorted heavy atoms chains). The range of the Pb-I bond lengths (*i.e.*, 3.0844(9) – 3.2606(4) Å) as well as the in-plane and out-of-plane distortion angles (see Table S2) are in agreement with the literature values (see, *f.e.*, [2], [3]). The crystal packing of the partial crystal structure consisted of slabs of couples of inorganic layers of corner-sharing PbI_6 octahedra (see Fig. S2), oriented along the longest axis, *i.e.*, the *b* axis (see Fig. S2b). The distance between the two nearest slabs of couples was ≈ 17.080 Å (see Figure S2b).

The monoclinic compound $(DA)_2(MA)_2Pb_3I_{10}$ (n = 3), in our knowledge, was not previously characterized. A view of the asymmetric unit and of its local environment is given in Figure 3b and 3c, respectively, together with the values of some bond angles listed in Table S3. The in-plane distortion angle Pb–I–Pb angle was 172.16° (see Figure and Table S3), The range of the Pb-I bond

lengths (*i.e.*, 3.0569(15) – 3.3037(13) Å) and the in-plane and out-of-plane distortion angles (see Table S3) are similar to literature values (see, *f.e.*, [2], [3]).

The crystal packing of the partial crystal structure was characterized by slabs of triplets of inorganic layers of corner-sharing PbI_6 octahedra (see Fig. 3), stacked along the longest axis, *i.e.*, the *a* axis (see Fig. 3d). The distance between the two nearest slabs of triplets was ≈ 17.105 Å (see Figure 3e).

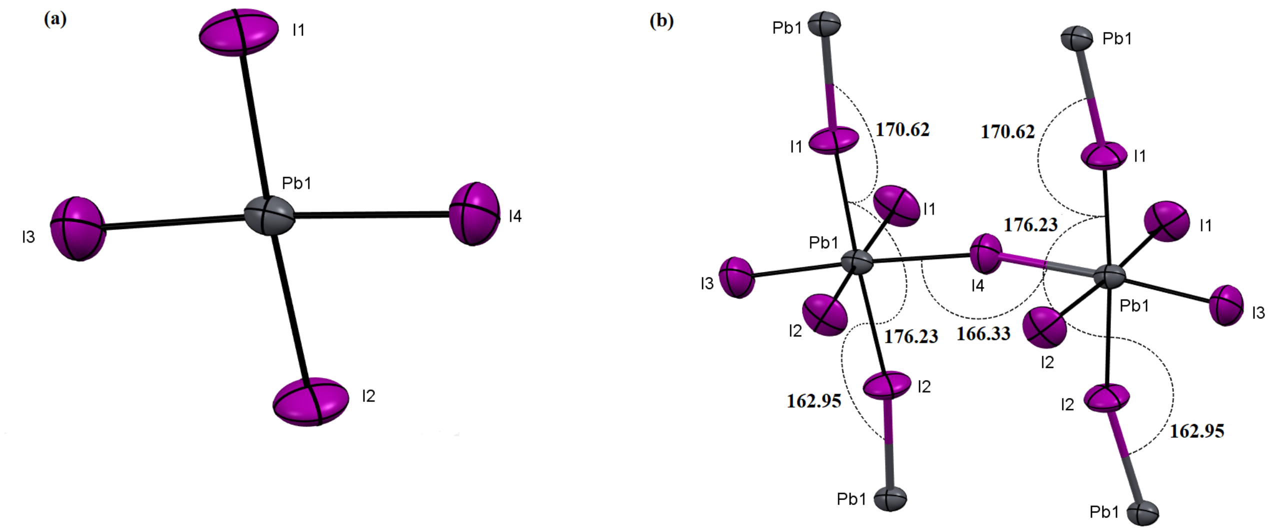

Figure S1 $(DA)_2(MA)Pb_2I_7$ (n = 2): (a) A view of the asymmetric unit with the atomic labelling scheme. (b) A view of the local environment of the asymmetric unit showing the polyhedral coordination of the Pb atom and the distortion angles (in °) of the inorganic layers (see Table S2). Ellipsoids are drawn at 50% of probability level.

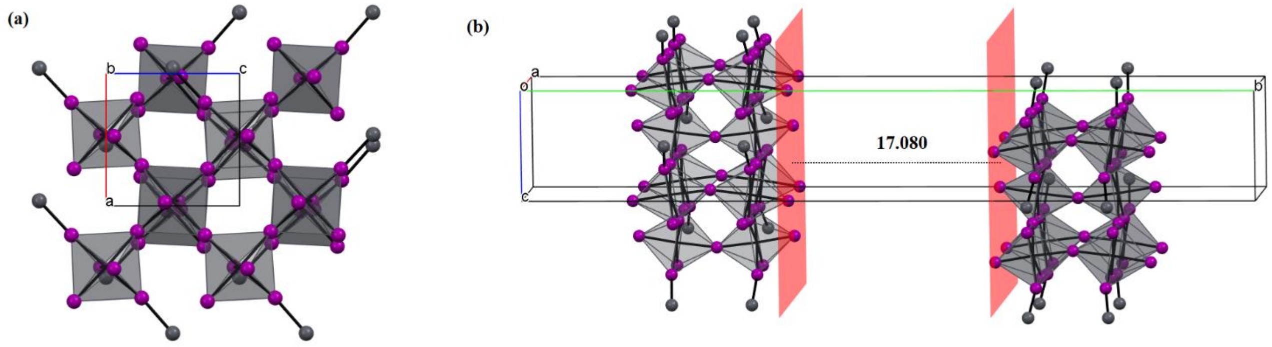

Figure S2 $(DA)_2(MA)Pb_2I_7$ (n = 2): (a) a view along *b* of the crystal packing; (b) a view of the crystal packing showing the distance (in Å) between the two nearest slabs of couples of inorganic layers.

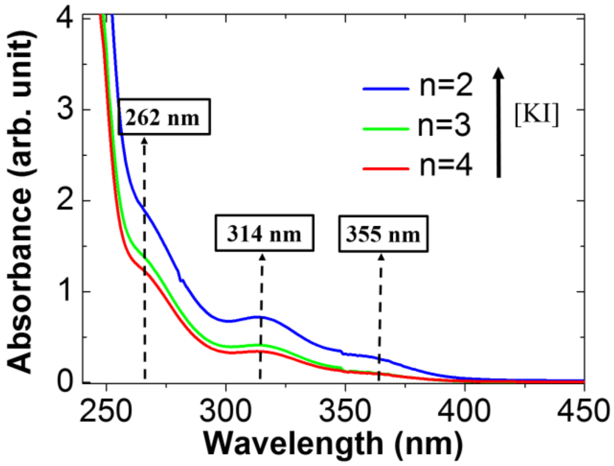

Figure S3: UV-vis adsorption spectra of solutions used for 2D-PVKs single crystals synthesis. To avoid signal saturation, solutions are much less concentrated compared to ones used for the synthesis, in particular: PbI_2 0.33 mM, MAI 1.48 mM, DAI 8.2 uM and KI 2.14 mM, 0.33 mM and 0.19 mM for n = 2, n = 3 and n = 4 respectively. The arrows point at the absorption maxima positions.

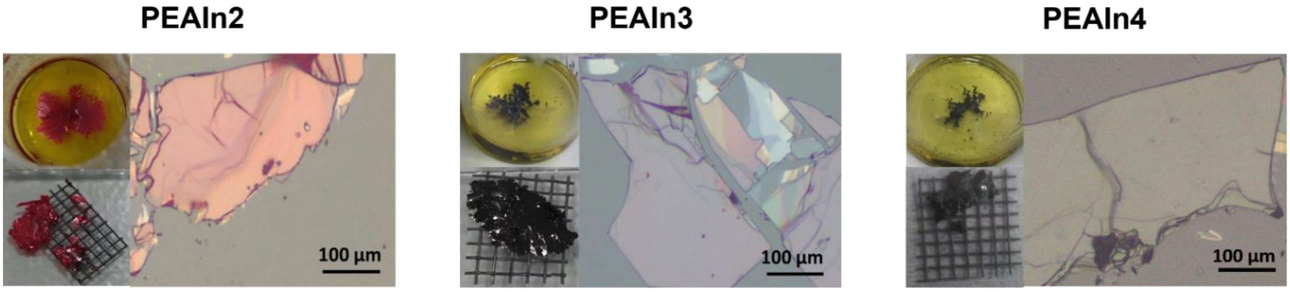

Figure S4: Photographs of 2D-PVKs single-crystalline flakes.

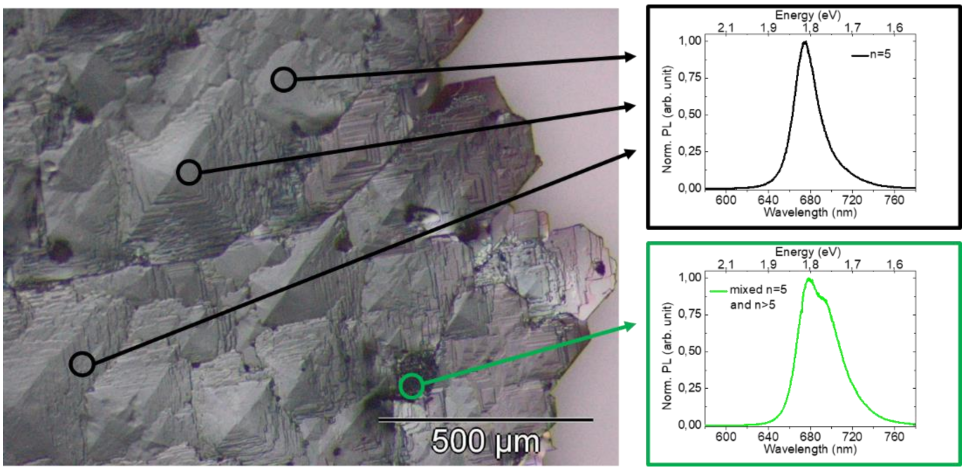

Figure S5: Optical microscope image and photoluminescence spectra taken focusing the laser on different regions of C12n5 crystals.

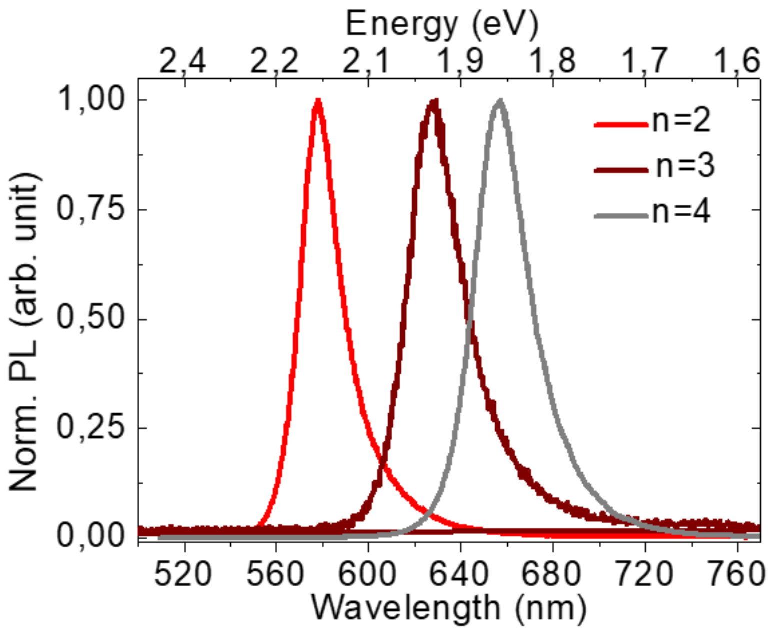

Figure S6: PL spectra of PEAIn2, PEAIn3 and PEAIn4 single-crystalline flakes at room temperature.

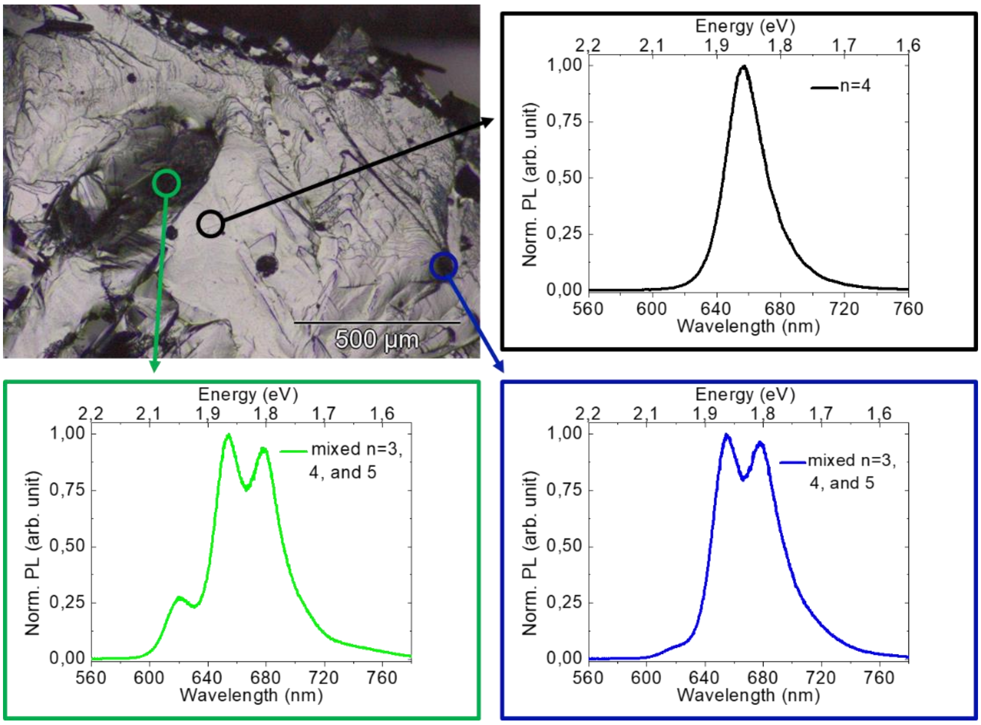

Figure S7: Optical microscope image and photoluminescence spectra taken focusing the laser on different regions of PEAI n = 4 crystals; From the photoluminescence analysis we can state that the dominant phase is n = 4 (PL peak at 660 nm) although when we explore extensively all the crystals we cannot exclude the presence of small n = 3 and n > 4 domains.

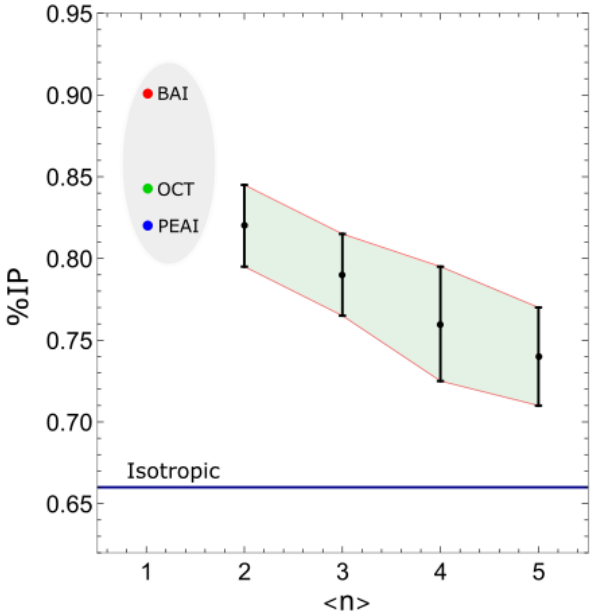

Figure S8: Estimated IP component for n = 2, n = 3, n = 4 and n = 5 C12 perovskite crystals, which shows a continuous decrease as n increases. The error bars are calculated considering several PL measurements collected in different spatial positions.

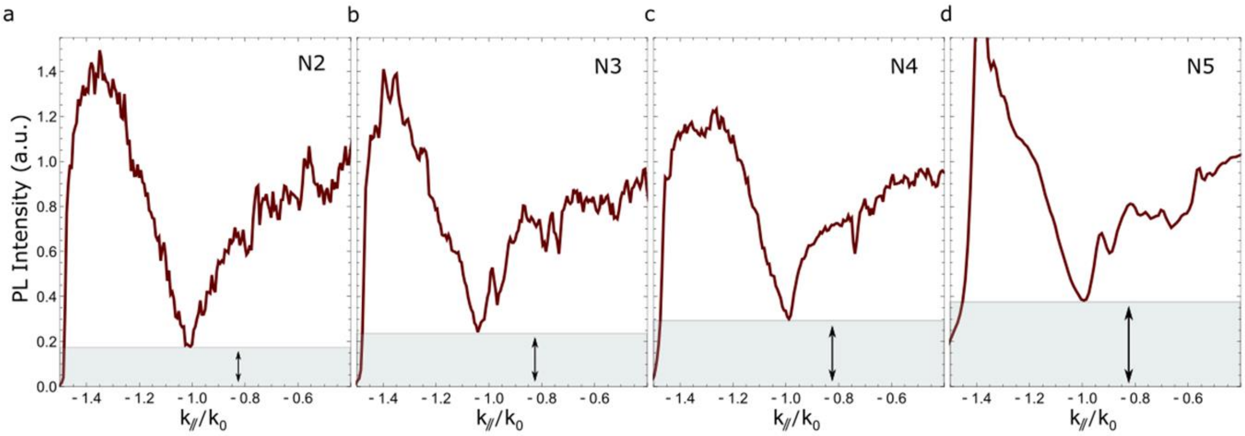

Figure S9: a), b), c), *p*-polarized Fourier-resolved photoluminescence profiles zoomed around the critical angle for C12 n2, n3, n4 and n5 respectively. The gray box and black arrow underline the different amplitude of the dip which gradually decreases as n increases. The OP component obtained from the fitting of the experimental data is 18% ± 3 for n = 2, 21% ± 3 for n = 3, 24% ± 4 for n = 4 and 26% ± 3 for n = 5 perovskite.

Table S1 Crystal data, data collection and structure refinement details for $(DA)_2(MA)Pb_2I_7$ (n = 2) and $(DA)_2(MA)_2Pb_3I_{10}$ (n = 3)

	**$(DA)_2(MA)Pb_2I_7$ (n = 2)**	**$(DA)_2(MA)_2Pb_3I_{10}$ (n = 3)**
Crystal data		
Chemical formula (only heavy atoms)	I_7Pb_2	$I_{10}Pb_3$
M_r	1302.70	1890.57
Crystal system, space group	Orthorhombic, *Pnma*	Monoclinic, $P2_1/c$
Temperature (K)	293	293
a, b, c (Å)<br>α, β, γ (°)	8.853(2), 59.264(3), 8.933(2)<br>90.0, 90.0, 90.0	36.026(3), 8.905(2), 8.862(2)<br>90.0, 91.06(2), 90.0
V (Å^3)	4686.8 (15)	2842.5 (9)
Z	4	2
Radiation type	Synchrotron, λ = 0.750 Å	Synchrotron, λ = 0.750 Å
μ (mm^{-1})	13.41	16.27
Crystal size (mm)	0.04 x 0.03 x 0.02	0.08 x 0.04 x 0.02
Data collection		
Diffractometer	BL-5A single axis goniometer	BL-5A single axis goniometer
Absorption correction	Multi-scan	Multi-scan
No. of measured, independent and observed [$I > 2\sigma(I)$] reflections	36766, 5750, 4433	43661, 6957, 5307
R_{int}	0.057	0.049
$(\sin \theta/\lambda)_{max}$ (Å^{-1})	0.667	0.667
Refinement		
$R[F^2 > 2\sigma(F^2)]$, $wR(F^2)$, S	0.064, 0.208, 1.06	0.094, 0.286, 1.04
No. of reflections	5750	6957
No. of parameters	43	61
$\Delta\rho_{max}$, $\Delta\rho_{min}$ (e Å^{-3})	4.03, -2.27	11.38, -2.04

Table S2 $(DA)_2(MA)Pb_2I_7$ (n = 2): main experimental and crystallographic details.

Crystal data	
I_7Pb_2	D_x = 1.846 Mg m^{-3}
M_r = 1302.70	Synchrotron radiation, λ = 0.750 Å
Orthorhombic, *Pnma*	Cell parameters from 8069 reflections
a = 8.853 (2) Å	θ = 0.72– 32.39°
b = 59.264 (3) Å	μ = 13.41 mm^{-1}
c = 8.933 (2) Å	*T* = 293 K
V = 4686.8 (15) Å^3	Plate, colour red
Z = 4	0.04 x 0.03 x 0.02 mm
F(000) = 2140.0	
Data collection	
BL-5A single axis goniometer diffractometer	4433 reflections with $I > 2\sigma(I)$
Radiation source: synchrotron	R_{int} = 0.057
ω scans, shutterless continuous rotation method	θ_{max} = 30.0°, θ_{min} = 1.5°
Absorption correction: multi-scan	*h* = -11→11
36766 measured reflections	*k* = -79→79
5750 independent reflections	*l* = -11→11
Refinement	
Refinement on F^2	43 parameters
Least-squares matrix: full	0 restraints
$R[F^2 > 2\sigma(F^2)]$ = 0.064	$w = 1/[\sigma^2(F_o^2) + (0.1221P)^2 + 14.933P]$ where $P = (F_o^2 + 2F_c^2)/3$
$wR(F^2)$ = 0.208	$(\Delta/\sigma)_{max}$ = 0.001
S = 1.06	$\Delta\rho_{max}$ = 4.03 e Å^{-3}
5750 reflections	$\Delta\rho_{min}$ = -2.27 e Å^{-3}
Special details	
Geometry. All estimated standard deviations (esds), except the esd in the dihedral angle between two least squares (l.s.) planes, are estimated using the full covariance matrix. The cell esds are taken into account individually in the estimation of esds in distances, angles and torsion angles; correlations between esds in cell parameters are only used when they are defined by crystal symmetry. An approximate (isotropic) treatment of cell esds is used for estimating esds involving l.s. planes.	

Fractional atomic coordinates and isotropic or equivalent isotropic displacement parameters (Å^2)

	x	*y*	*z*	$U_{iso}*/U_{eq}$
Pb1	0.46131 (4)	0.19537 (2)	0.00089 (3)	0.04861 (16)
I1	0.22111 (10)	0.19154 (2)	-0.25978 (10)	0.0994 (3)

I4	0.46196 (17)	0.2500	-0.04256 (19)	0.0901 (4)
I2	0.69451 (10)	0.20248 (2)	0.26658 (10)	0.0969 (3)
I3	0.47496 (15)	0.14410 (2)	0.05866 (14)	0.1061 (4)

Atomic displacement parameters (Å^2)

	U^{11}	U^{22}	U^{33}	U^{12}	U^{13}	U^{23}
Pb1	0.0416 (2)	0.0665 (3)	0.0377 (2)	0.00088 (12)	0.00017 (10)	0.00183 (11)
I1	0.0798 (5)	0.1419 (8)	0.0764 (5)	0.0113 (5)	-0.0450 (4)	-0.0120 (4)
I4	0.1189 (11)	0.0562 (5)	0.0954 (8)	0.000	0.0010 (7)	0.000
I2	0.0793 (5)	0.1322 (7)	0.0791 (5)	0.0074 (5)	-0.0468 (4)	-0.0059 (5)
I3	0.1622 (10)	0.0613 (4)	0.0948 (7)	0.0048 (5)	-0.0007 (6)	0.0040 (4)

Geometric parameters (Å, º)

Pb1—I3	3.0844 (9)	Pb1—I4	3.2605 (4)
Pb1—I1^{i}	3.1592 (9)	I1—Pb1^{iii}	3.1592 (9)
Pb1—I1	3.1616 (9)	I4—Pb1^{iv}	3.2606 (4)
Pb1—I2^{ii}	3.1735 (9)	I2—Pb1^{v}	3.1735 (9)
Pb1—I2	3.1738 (9)		
I3—Pb1—I1^{i}	90.85 (3)	I2^{ii}—Pb1—I2	88.69 (2)
I3—Pb1—I1	94.52 (3)	I3—Pb1—I4	176.34 (4)
I1^{i}—Pb1—I1	88.992 (19)	I1^{i}—Pb1—I4	89.36 (4)
I3—Pb1—I2^{ii}	92.88 (3)	I1—Pb1—I4	89.13 (4)
I1^{i}—Pb1—I2^{ii}	176.07 (3)	I2^{ii}—Pb1—I4	86.99 (4)
I1—Pb1—I2^{ii}	89.49 (3)	I2—Pb1—I4	87.49 (4)
I3—Pb1—I2	88.86 (3)	Pb1^{iii}—I1—Pb1	170.62 (4)
I1^{i}—Pb1—I2	92.62 (3)	Pb1—I4—Pb1^{iv}	166.33 (6)
I1—Pb1—I2	176.24 (3)	Pb1^{v}—I2—Pb1	162.95 (4)

Symmetry codes: (i) *x*+1/2, *y*, -*z*-1/2; (ii) *x*-1/2, *y*, -*z*+1/2; (iii) *x*-1/2, *y*, -*z*-1/2; (iv) *x*, -*y*+1/2, *z*; (v) *x*+1/2, *y*, -*z*+1/2.

Table S3 $(DA)_2(MA)_2Pb_3I_{10}$ (n = 3): main experimental and crystallographic details.

Crystal data	
$I_{10}Pb_3$	$F(000) = 1552$
$M_r = 1890.57$	$D_x = 2.209$ Mg m^{-3}
Monoclinic, $P2_1/c$	Synchrotron radiation, $\lambda = 0.750$ Å
$a = 36.026$ (3) Å	Cell parameters from 11346 reflections
$b = 8.905$ (2) Å	$\theta = 0.60$– 35.87°
$c = 8.862$ (2) Å	$\mu = 16.27$ mm^{-1}
$\beta = 91.06$ (2)°	$T = 293$ K
$V = 2842.5$ (9) Å^3	Plate, colour red
$Z = 2$	0.08 x 0.04 x 0.02 mm
Data collection	
BL-5A single axis goniometer diffractometer	5307 reflections with $I > 2\sigma(I)$
Radiation source: synchrotron	$R_{int} = 0.049$
ω scans, shutterless continuous rotation method	$\theta_{max} = 30.0°$, $\theta_{min} = 1.2°$
Absorption correction: multi-scan	$h = -48 \rightarrow 48$
43661 measured reflections	$k = -11 \rightarrow 11$
6957 independent reflections	$l = -11 \rightarrow 11$
Refinement	
Refinement on F^2	61 parameters
Least-squares matrix: full	0 restraints
$R[F^2 > 2\sigma(F^2)] = 0.094$	$w = 1/[\sigma^2(F_o^2) + (0.1731P)^2 + 42.5881P]$ where $P = (F_o^2 + 2F_c^2)/3$
$wR(F^2) = 0.286$	$(\Delta/\sigma)_{max} < 0.001$
$S = 1.04$	$\Delta\rho_{max} = 11.38$ e Å^{-3}
6957 reflections	$\Delta\rho_{min} = -2.04$ e Å^{-3}
Special details	
Geometry. All estimated standard deviations (esds), except the esd in the dihedral angle between two least squares (l.s.) planes, are estimated using the full covariance matrix. The cell esds are taken into account individually in the estimation of esds in distances, angles and torsion angles; correlations between esds in cell parameters are only used when they are defined by crystal symmetry. An approximate (isotropic) treatment of cell esds is used for estimating esds involving l.s. planes.	

Fractional atomic coordinates and isotropic or equivalent isotropic displacement parameters (Å^2)

	x	y	z	U_{iso}*/U_{eq}
Pb1	0.0000	0.0000	0.5000	0.0423 (2)
Pb2	0.17896 (2)	-0.00143 (5)	0.51233 (6)	0.0426 (2)
I1	0.08747 (3)	0.0238 (2)	0.50981 (18)	0.0845 (5)
I2	0.16733 (5)	-0.24246 (17)	0.76927 (17)	0.0874 (5)
I3	0.18376 (5)	0.25461 (17)	0.75785 (16)	0.0909 (5)

I4	0.00403 (5)	-0.20400 (17)	0.20337 (16)	0.0826 (5)
I5	0.26256 (4)	-0.0593 (2)	0.5066 (3)	0.1056 (6)

Atomic displacement parameters (Å^2)

	U^{11}	U^{22}	U^{33}	U^{12}	U^{13}	U^{23}
Pb1	0.0519 (4)	0.0381 (4)	0.0369 (4)	0.0016 (3)	0.0016 (3)	0.0001 (2)
Pb2	0.0567 (4)	0.0358 (3)	0.0356 (3)	0.00126 (19)	0.0034 (2)	-0.00008 (16)
I1	0.0426 (6)	0.1064 (12)	0.1044 (13)	0.0011 (6)	0.0014 (7)	-0.0007 (8)
I2	0.1264 (12)	0.0692 (8)	0.0671 (8)	0.0129 (8)	0.0157 (7)	0.0383 (6)
I3	0.1440 (14)	0.0650 (8)	0.0641 (8)	-0.0097 (8)	0.0147 (8)	-0.0342 (6)
I4	0.1177 (11)	0.0659 (8)	0.0645 (7)	-0.0096 (7)	0.0120 (7)	-0.0344 (6)
I5	0.0508 (7)	0.0932 (12)	0.1730 (19)	0.0057 (7)	0.0084 (8)	-0.0021 (12)

Geometric parameters (Å, º)

Pb1—I1^{i}	3.1577 (12)	Pb2—I3^{iv}	3.1561 (13)
Pb1—I1	3.1578 (12)	Pb2—I2^{v}	3.1597 (13)
Pb1—I4^{ii}	3.1949 (12)	Pb2—I2	3.1627 (13)
Pb1—I4^{iii}	3.1949 (12)	Pb2—I1	3.3037 (13)
Pb1—I4	3.2009 (12)	I2—Pb2^{ii}	3.1596 (13)
Pb1—I4^{i}	3.2009 (12)	I3—Pb2^{vi}	3.1561 (13)
Pb2—I5	3.0569 (15)	I4—Pb1^{vii}	3.1949 (12)
Pb2—I3	3.1541 (13)		
I1^{i}—Pb1—I1	180.0	I3—Pb2—I3^{iv}	89.23 (2)
I1^{i}—Pb1—I4^{ii}	89.71 (5)	I5—Pb2—I2^{v}	89.16 (6)
I1—Pb1—I4^{ii}	90.29 (5)	I3—Pb2—I2^{v}	175.50 (5)
I1^{i}—Pb1—I4^{iii}	90.29 (5)	I3^{iv}—Pb2—I2^{v}	91.37 (5)
I1—Pb1—I4^{iii}	89.71 (5)	I5—Pb2—I2	92.37 (6)
I4^{ii}—Pb1—I4^{iii}	180.0	I3—Pb2—I2	90.01 (5)
I1^{i}—Pb1—I4	89.98 (5)	I3^{iv}—Pb2—I2	175.41 (5)
I1—Pb1—I4	90.02 (5)	I2^{v}—Pb2—I2	89.04 (2)
I4^{ii}—Pb1—I4	89.585 (19)	I5—Pb2—I1	174.04 (5)
I4^{iii}—Pb1—I4	90.415 (19)	I3—Pb2—I1	89.85 (5)
I1^{i}—Pb1—I4^{i}	90.02 (5)	I3^{iv}—Pb2—I1	90.89 (5)
I1—Pb1—I4^{i}	89.98 (5)	I2^{v}—Pb2—I1	85.68 (5)
I4^{ii}—Pb1—I4^{i}	90.415 (19)	I2—Pb2—I1	84.58 (5)
I4^{iii}—Pb1—I4^{i}	89.585 (19)	Pb1—I1—Pb2	172.16 (7)
I4—Pb1—I4^{i}	180.0	Pb2^{ii}—I2—Pb2	164.37 (7)
I5—Pb2—I3	95.28 (6)	Pb2—I3—Pb2^{vi}	173.37 (7)
I5—Pb2—I3^{iv}	92.21 (6)	Pb1^{vii}—I4—Pb1	158.32 (6)

Symmetry codes: (i) -*x*, -*y*, -*z*+1; (ii) *x*, -*y*-1/2, *z*+1/2; (iii) -*x*, *y*+1/2, -*z*+1/2; (iv) *x*, -*y*+1/2, *z*-1/2; (v) *x*, -*y*-1/2, *z*-1/2; (vi) *x*, -*y*+1/2, *z*+1/2; (vii) -*x*, *y*-1/2, -*z*+1/2.

Computing details

Program used for cell refinement and data reduction *XDS* [4]; program used to solve structure: *SIR2019* [5]; program used to refine structure *SHELXL2014/7* [6]; molecular graphics: *Mercury* [7]; software used to prepare material for publication: *WinGX* [8] and *publCIF* [9].

Table S4

	**PbI_2/MAI/C12 molar ratio**	**KI/PbI_2**	**PbI_2 [M]**	**MAI [M]**	**C12 [M]**	**KI [M]**	**RPP synthesized**
**C12**	1/4.5/0.025	6.5:1	0.47	2.13	0.01	3.07	n = 2
	1/4.5/0.025	1:1	0.47	2.13	0.01	0.47	n = 3
	1/4.5/0.025	0.6:1	0.47	2.13	0.01	0.29	n = 4
	1/4.5/0.025	0.57:1	0.47	2.13	0.01	0.27	n = 5 *
	1/4.5/0.025	0.55:1	0.47	2.13	0.01	0.26	no perovskite synthesized (precipitation of lead derivatives)
	1/4.5/0.025	0.49:1	0.47	2.13	0.01	0.23	no perovskite synthesized
	**Modulation of PbI_2/MAI ratio**						
	1/4.3/0.025	0.6:1	0.47	2.03	0.01	0.29	n = 3 and n > 3
	1/3.75/0.025	0.6:1	0.47	1.77	0.01	0.29	n = 3 and n ≠ 3
	1/4.4/0.025	0.6:1	0.47	2.08	0.01	0.29	n = 4 and n = 5
	1/3/0.025	2.5:1	0.47	1.41	0.01	1.17	n = 2 and n > 2

* n = 5 dominant (presence of very few n = 4 domains)

Table S5

	**PbI_2/MAI/PEAI molar ratio**	**KI/PbI_2**	**PbI_2 [M]**	**MAI [M]**	**PEAI [M]**	**KI [M]**	**RPP synthesized**
**PEAI**	1/2.4/0.2	7.5:1	0.64	1.55	0.12	4.81	n = 2
	1/2.4/0.2	6.2:1	0.64	1.55	0.12	4.01	n = 3
	1/2.4/0.2	5.4:1	0.64	1.55	0.12	3.51	n = 4 *
	1/2.4/0.2	4.3:1	0.64	1.55	0.12	2.81	n = 4*
	**Modulation of PbI_2/MAI ratio**						
	1/2.8/0.2	5.6:1	0.64	1.81	0.12	3.61	n = 3, n = 4, n = 5
	1/2.8/0.2	5:1	0.64	1.81	0.12	3.21	n = 4 and n > 4

* n = 4 dominant (presence of very few n = 3 and n = 5 domains)

References

[1] M. Cinquino, L. Polimeno, G. Lerario, M. De Giorgi, A. Moliterni, V. Olieric, A. Fieramosca, S. Carallo, R. Mastria, V. Ardizzone, L. Dominici, D. Ballarini, C. Giannini, D. Sanvitto, A. Rizzo, L. De Marco, J. Lumin. 2020, 221, 117079.
[2] C. C. Stoumpos, D. H. Cao, D. J. Clark, J. Young, J. M. Rondinelli, J. I. Jang, J. T. Hupp, M. G. Kanatzidis, Chem. Mater. 2016, 28, 2852.
[3] I. Spanopoulos, I. Hadar, W. Ke, Q. Tu, M. Chen, H. Tsai, Y. He, G. Shekhawat, V. P. Dravid, M. R. Wasielewski, A. D. Mohite, C. C. Stoumpos, M. G. Kanatzidis, J. Am. Chem. Soc. 2019, 141, 5518.
[4] W. Kabsch, Acta Crystallogr. Sect. D 2010, 66, 125.
[5] M. C. Burla, R. Caliandro, B. Carrozzini, G. L. Cascarano, C. Cuocci, C. Giacovazzo, M. Mallamo, A. Mazzone, G. Polidori, J. Appl. Cristallogr. 2015, 48, 306.
[6] G. M. Sheldrick, Acta Crystallogr. Sect. A 2015, A71, 3.
[7] C. F. Macrae, I. Sovago, S. J. Cottrell, P. T. A. Galek, E. Pidcock, M. Platings, G. P. Shields, J. S. Stevens, M. Towler, P. A. Wood, J. Appl. Cristallogr. 2020, 53, 226.
[8] L. J. Farrugia, J. Appl. Cryst 2012, 45, 849.
[9] S. P. Westrip, Appl. Crystallogr. 2010, 43, 920.
